# The prognostic potential of coilin in association with p27 expression in pediatric acute lymphoblastic leukemia for disease relapse

**DOI:** 10.1186/s12935-018-0600-5

**Published:** 2018-07-27

**Authors:** Zhi-Xia Yue, Rui-qi Gao, Chao Gao, Shu-Guang Liu, Xiao-Xi Zhao, Tian-Yu Xing, Jing Niu, Zhi-Gang Li, Hu-Yong Zheng, Wei Ding

**Affiliations:** 1 Department of Medical Genetics and Developmental Biology, School of Basic Medical Sciences, Capital Medical University, Beijing, 100069 China; 2 Beijing Key Laboratory of Pediatric Hematology Oncology, National Key Discipline of Pediatrics, Ministry of Education, MOE Key Laboratory of Major Diseases in Children, Hematology Oncology Center, Beijing Children’s Hospital, Capital Medical University, National Center for Children’s Health, Beijing, 100045 China; 3 Department of Biochemistry and Molecular Biology, School of Basic Medical Sciences, Capital Medical University, Beijing, 100069 China; 4 Beijing Key Laboratory for Tumor Invasion and Metastasis Research, Cancer Institute of Capital Medical University, Beijing, 100069 China; 5 Key Laboratory of Major Diseases in Children (Capital Medical University), Ministry of Education, National Key Discipline of Pediatrics, Ministry of Education, Hematology Center, Beijing Children’s Hospital, Capital Medical University, Beijing, 100045 China

**Keywords:** Acute lymphoblastic leukemia, Pediatrics, Chemotherapy, Coilin, p27, Relapse

## Abstract

**Background:**

Cajal body (CB) is a nucleic organelle where small nuclear ribonucleoproteins undergo modification, maturation, splicing and/or assembly. Coilin is the marker structural protein of CBs. The expression level and cellular localization of coilin is sensitive to chemotherapeutic reagents, such as cisplatin. The gene of cyclin-dependent kinase inhibitor 1B (p27) is located with a high incidence translocation region of leukemic chromosomes, and its expression was of prognosis values in a variety of adult leukemia types. The exact profile and associated functions of coilin, as well as p27, in children’s acute lymphoblastic leukemia (ALL) is obscure.

**Methods:**

Bone marrow samples from 144 patients with ALL were collected. The expression levels of coilin and p27 were detected by qRT-PCR. The patient cohort was divided into low and high groups of coilin and p27 respectively. The prognosis and clinicobiological characteristics of different groups were investigated, especially focused on the treatment outcome. Leukemia cells of Reh or RS4;11 were exposed to different concentrations of DNR, prior to the detection for morphological changes of coilin by immunofluorescence. In Reh cells, lentivirus sh-coilin was used to silence coilin expression. Western blotting was used to detect coilin and p27 expression; flow cytometry was used for cell cycle and apoptosis assay; MTS method was used for measuring cell viability to examine the drug sensitivity of DNR.

**Results:**

In this study, we found that daunorubicin was able to induce significant morphological changes of CBs in Reh and RS4;11 cells. Knockdown the expression of coilin increased the sensitivity to daunorubicin and inhibited the expression of p27 in Reh cells, and led to increased apoptosis. Importantly, not only the levels of coilin and p27 mRNA expression at initial diagnosis ALL children are markedly higher than those at complete remission (CR), but also both coilin and p27 expression in the relapsed patients was observed significantly higher comparing to the continuous CR patients. The 4-year EFS and RFS indicated that low levels of both coilin and p27 group favored better prognosis (p < 0.05).

**Conclusions:**

Our results indicated that consideration of coilin and p27 levels could be a prognostic reference for predicting the outcome of pediatric ALL patients, especially for disease recurrence. Reduction of coilin expression was sufficient to increase the sensitivity of leukemic cells to daunorubicin treatments, and during which possibly involved functions of p27 in cell cycle regulation and its effects on cell apoptosis.

## Background

Acute lymphoblastic leukemia (ALL) is the most common malignancy in children worldwide [1]. The chemotherapy regimen for ALL patients involving multiple drugs combinations has developed and constantly being optimized based on risk classification and stratification [2]. Although the outcome following treatments of pediatric ALL has dramatically improved during the past two decades, there still remained about 15% of patients failed the chemotherapy. The relapse of pediatric ALL has become a bottleneck, as well as a problematic challenge for the further improvement in ALL treatments. In clinics, without prognostic indications of ALL relapse, physicians often faced a dilemma about whether to recommend chemotherapy dose reinforcement for patients with attenuated relieves [3–5].

Among the current effective chemotherapeutic regimens, ten or more different drugs were selected and prescribed to treat childhood ALL for over 2–3 years periods [6]. Daunorubicin (DNR) is one of the most frequently used component, especially being used for treating pediatric ALL. DNR is a potent topoisomerase II inhibitor and is able to cause the accumulation of DNA double-strand breaks (DSBs) [7]. The ability of DNR to induce massive apoptosis in leukemia cells has made it one of the most effective drugs within a chemotherapy regimen. Unfortunately, the drug resistance to DNR not only directly contributed to the failure of chemotherapy, but also can be a major cause for the disease relapse [8]. The molecular mechanism for the development of drug resistance can be extremely sophisticated, including the enhanced DNA damage repair capacity, tolerance to apoptosis, increased energy-dependent drug efflux and changes in drug metabolism, etc. [9]. Among various enzymes and proteins related to chemoresistance, glutathione transferase and topoisomerase II are the ones that has been early recognized. Studies have shown that topoisomerase II was also an important molecule for the maintenance of nuclear structure and chromatin configuration [10]. The abnormality in topoisomerase II expression levels may lead to observable changes in cell nuclei through microscopy.

Cajal bodies (CB) is a common nuclear structure observed in different cell types that recently rediscovered for its important biological functions [11]. The morphology of CB rapidly responds and is extremely sensitive to DNA damages [12]. Coilin is a hallmark protein of the Cajal bodies, and primarily been used to monitor the dynamics of CB structures. Coilin is also functionally involved in many CB associated nuclear activities, including the biogenesis of small nuclear ribonucleoproteins (snRNPs), small Cajal body-specific RNPs (scaRNPs), small nucleolar RNPs (snoRNPs), as well as the processing of histone pre-mRNAs at the 3′-ends [13]. It has been reported that the number counts and the shapes or sizes of CBs were subjected to significant changes when cells were exposed to a wide array of DNA damage reagents, such as cisplatin, etoposide, UV-C or gamma irradiation [14]. Researches have also found that coilin was indeed play a role in the DNA damage responses. For examples, coilin could directly interact with Ku protein to inhibit non-homologous end joining (NHEJ) during DNA repair process [15]. TCAB1, another essential CB component, accumulated at DNA damage sites in an ATM/H2AX/MDC1-dependent manner and enhanced DNA repair via homologous recombination (HR) and NHEJ when cells treated with micro-irradiation [16]. Increasing evidence supported the possibility that coilin was in association with drug sensitivity in chemotherapy due to its role in the modulation of cellular DNA damage/repair pathways.

Latest reports showed that coilin^−/−^ mice exhibited reduced viability as a consequence of defects in snRNP biogenesis and splicing. Additional studies have shown that the knockout of coilin in zebrafish led to immediate death of the embryos [17, 18]. The knockdown of coilin in U2OS cells with etoposide treatments, DNA damage responses increased γH2AX at the protein levels, subsequently led to substantial phenotypical changes in cell growth and cell cycle arrest at the S phase and G2/M phase [19]. The housekeeping protein of p27 is an important cell cycle regulatory molecule which negatively controls cell-cycle progression. The human p27 gene is located at human chromosome 12p13 [20]. This genome region has been previously identified as a common site of chromosomal translocation in ALL.

Different from the majority of human solid tumors, the expression of p27 at both mRNA and protein levels were much higher than normal tissues, especially in chronic lymphocytic leukemia [21]. Patients with low p27 mRNA expression were found to have a better survival outcome than those with intermediate/high expression patients [22]. In pediatric ALL, the expression of p27 remained to be obscure from the literature. There is also no clinical report summarizing the potential role of either p27 or coilin in the sensitivity of chemotherapy, especially in connection with the DNA damage and repair pathways. In this present study, we investigated the effect of coilin knockdown on the expression of p27 and the sensitivity to daunorubicin in ALL cells. The clinical significance of coilin and p27 in the initial diagnosis and retrospection of relapsed pediatric ALL were explored.

## Methods

### Patient information

A total of 144 children (from 5 months to 14.9 years old with a median age of 4.1 years) with newly diagnosed BCP-ALL between October 2011 and December 2012 in Beijing Children’s Hospital (BCH) were enrolled in this study. All patients were diagnosed based on comprehensive indexes of morphology, immunology, cytogenetics and molecular biology (MICM). And all of them were treated with the protocol established by Chinese Children’s Leukemia Group in 2008. The details in risk stratification and treatment of this protocol has been described previously [3]. G-banding karyotyping and multiplex reverse transcription polymerase chain reaction (RT-PCR) were used to identify the genetic subtypes of leukemia.

In this study, to ensure the leukemic cells existed as the main component in the bone marrow (BM) samples, the inclusion criterion for patient admission was set as ≥ 70% leukemic cells in the BM samples at diagnosis. Paired BM samples in 22 patients were collected at the time of initial diagnosis and in complete remission (CR) at day 78 (before consolidation therapy) respectively. The clinical characteristics of these patients were shown in Table 1. BM samples obtained from the 5 idiopathic thrombocytopenic purpura (ITP) patients were used as negative control.

**Table 1:** Clinical information and statistics about the included 144 patients

Characteristics	Total (n %)
Gender	
Male	86 (59.7)
Female	58 (40.3)
Age (years)	
1–9	124 (86.1)
≥ 10	20 (13.9)
Median	4.1
Range	0.5–14.9
WBC (× 10^9^/l)	
< 50	123 (85.4)
≥ 50	21 (14.6)
Median	8.3
Range	1.1–990.0
CNS involvement	
No	143 (99.3)
Yes	1 (0.7)
Fusion gene	
*TEL/AML1*	30 (20.8)
*E2A/PBX1*	8 (5.6)
*BCR/ABL1*	10 (6.9)
*MLL* rearrangement	6 (4.2)
Others	2 (1.4)
Prednisone response	
Good	137 (95.1)
Poor	7 (4.9)
MRD at day 33	
< 1 × 10^−4^	38 (26.4)
≥ 1 × 10^−4^	106 (73.6)
Treatment outcome	
Relapse	13 (9.1)
Death	11 (7.6)
CCR	120 (83.3)
Survival (%)	
4-year EFS	83.1 ± 3.1

*WBC* white blood cell, *CNS* central nervous system, *MRD* minimal residual disease, *CCR* continuous complete remission, *EFS* event-free survival

This research was approved by the BCH Institutional Ethics Committee. Informed consents were obtained from the parents or guardians of each case according to the Declaration of Helsinki.

### Cell culture, lentivirus infection and chemical treatments

Reh and RS4;11 cells were purchased from the cell bank at Peking Union Medical University. The Reh cell line was detected for unique STR profiles in China and confirmed with the correct genetic background. Cells were expanded and cultured in Roswell Park Memorial Institute (RPMI) with l-glutamine (Hyclone, Beijing, China) supplemented with 10% fetal bovine serum (FBS) (Invitrogen, Carlsbad, CA). For lentivirus infection experiments, cells were seeded in 6- or 12-well plates. The lentivirus carrying a sh-coilin cassette (4 × 10^8^ TU/ml) was purchased from Shanghai Genechem Co., Ltd, China. The targeting RNAi sequence of sh-coilin and a control scramble were as GAGAGAACCTGGGAAATTT and TTCTCCGAACGTGTCACGT respectively.

Daunorubicin (DNR, Sigma-Aldrich, USA) was used at 0.01, 0.03 and 0.05 μg/ml for 24 h. Arabinocytidine, Vincristine and Adriamycin were purchased from Dalian Meilun Biotechnology Co., Ltd, China.

### RNA isolation, reverse transcription, and quantitative real-time PCR

Mononuclear cells from the BM samples were isolated by Ficoll–Hypaquedensity-gradient centrifugation and then preserved at − 80 °C. The total RNAs were extracted from cells (frozen at − 80 °C) using Trizol reagent (Invitrogen, Paisley, UK) according to the standard protocol. The RNA were transcribed reversely into cDNAs using random hexamers and Moloney murine leukemia virus (MMLV) reverse transcriptase (Promega, Madison, USA) according to the manufacturer’s instructions. The coilin and p27 expression were measured by real-time quantitative RT-PCR (qRT-PCR) using an Applied Biosystems ViiA7 (Life Technologies, USA) with the TaqMan probes for human coilin mRNA (assay ID Hs00982300_m1) or p27 mRNA (assay ID Hs01597588_m1). The beta-glucuronidase (GUS) gene was used as the internal control. The primer sequences used for GUS were forward: 5′-GAAAATATGT GGTTGGAGAG CTCATT-3′ and reverse: 5′-CCGAGTGAAG ATCCCCTTTT TA-3′.

The PCR reactions were performed in a total volume of 10 μl, including 5 μl TaqMan gene expression master mix (Applied Biosystems, USA), 0.5 μl Gene Expression Assay probe, 1 μl cDNA and 3.5 μl deionized water. The PCR program was carried out at 95 °C for 10 min, followed by 50 cycles of 95 °C for 15 s and 60 °C for 60 s. Each sample was detected in triplicates. The relative levels of coilin and p27 were determined by 2^−△△CT^ method.

### Immunofluorescence microscopy

Cells were grown on a 12-well plates and treated with daunorubicin (DNR) for 24 h. Before fixation, the cells were transferred onto glass coverslips in another 12-well plate (treated with poly-l-lysine in advance for 30 min). The cells were then treated as follows: fixed in 4% paraformaldehyde (Sigma-Aldrich) for 15 min at room temperature, washed with PBS for 5 min × 3, permeabilized with 0.5% Triton X-100 for 10 min, blocked with 2.0% bovine serum albumin (BSA, Cat. No. 0332, USA) for 1 h, and incubated with coilin primary antibody (1:50 dilution, sc-32860, Santa Cruz, CA, USA) overnight at 4 °C and subsequently suitable secondary antibodies (1:200 dilution, Life, Carlsbad, CA, USA). The nucleus was stained with DAPI (40, 6-diamidino-2-phenylindole, Cat. No. ZLI-9557; ZhongShan JinQiao, Beijing, China). Fluorescence images were collected with a confocal microscope Leica TCS SP5 MP system.

### Western blotting

Cells grown in six-well plates were harvested on ice and lysed in 100 μl RIPA buffer (Thermo, Rockford, IL, USA) containing the full cocktail of protease inhibitors (Thermo). The protein concentrations were determined using the BCA protein assay kit (Novagen, San Diego, CA, USA). The loaded samples containing 30 μg protein per lane were separated on 12% SDS-PAGE gels and then transferred onto Polyvinylidene Fluoride (PVDF) filters (Millipore, Billerica, MA, USA). After blocking with 1.5% (m/v) BSA in TBST for 1 h at room temperature, the proteins on PVDF were blotted with rabbit polyclonal anti-coilin (sc-32860, 1:1000, Santa Cruz, CA, USA) or rabbit anti-p27 (sc-528, 1:1000, Santa Cruz, CA, USA) specific antibodies, mouse monoclonal anti-phospho-Histone H2AX (Ser139) (05-636, 1:1000, Millipore, USA) or HRP-conjugated mouse anti-GAPDH (KC-5G5, 1:1000, Kangchen-BioTech, Shanghai, China) antibodies. After washing three times with TBST for 10 min each, the blots were incubated with appropriate secondary antibodies at room temperature for 1 h (1:10,000, Signalway Antibody LLC, USA). The proteins were visualized with an enhanced chemiluminescence (ECL) kit (Millipore, USA) and imaged under a detection system.

### Cell viability assay (MTS method)

The cell viability assays were performed using the CellTiter-96 Aqueous-One Solution Cell Proliferation (MTS) Assay kit (G3580, Promega, USA). Briefly, 20 μl of MTS reagent was added into the 96-well plates per well, and incubated (37 °C, 5% CO_2_) for 24 h. The plates were then loaded into the ELx808™ Absorbance Microplate Reader (BioTek, Winooski, VT, USA) to measure the absorbance at 490 nm.

### Apoptosis assay

For apoptosis studies, a PE Annexin V Apoptosis Detection Kit I (4289813, eBioscience, USA) was used. The Reh cells infected with control or sh-coilin for 96 h were harvested and washed with a binding buffer. Then, the cells were stained with 5 μl of Annexin V-PE for 15 min at room temperature in dark. Wash twice with the binding buffer, the samples were stained with 5 μl of 7AAD before detection. The cellular apoptosis were analyzed also using a Gallios Flow Cytometer (Beckman Coulter).

### Statistical analysis

The statistical tests were performed using SPSS 16.0 software for data analyses. The date of May 30, 2016 was set as the reference date for the terminating dead line of data collection. Comparisons between the subgroups for this study were evaluated by non-parametric tests. Event-free survival (EFS) was estimated from the date of diagnosis to the date meeting one of the following events: induction failure, relapse, death or the last contact with patients in continuous CR. Relapse-free survival (RFS) was defined from the date of diagnosis to the date of disease relapse. The significance in RFS and EFS were estimated with the Kaplan–Meier survival analysis. All tests with a *p *< 0.05 in two-sided distributions were considered statistically significant.

## Results

### Daunorubicin-induced DNA damage disrupted the nuclear localization of coilin in leukemia cells

**Fig. 1:**
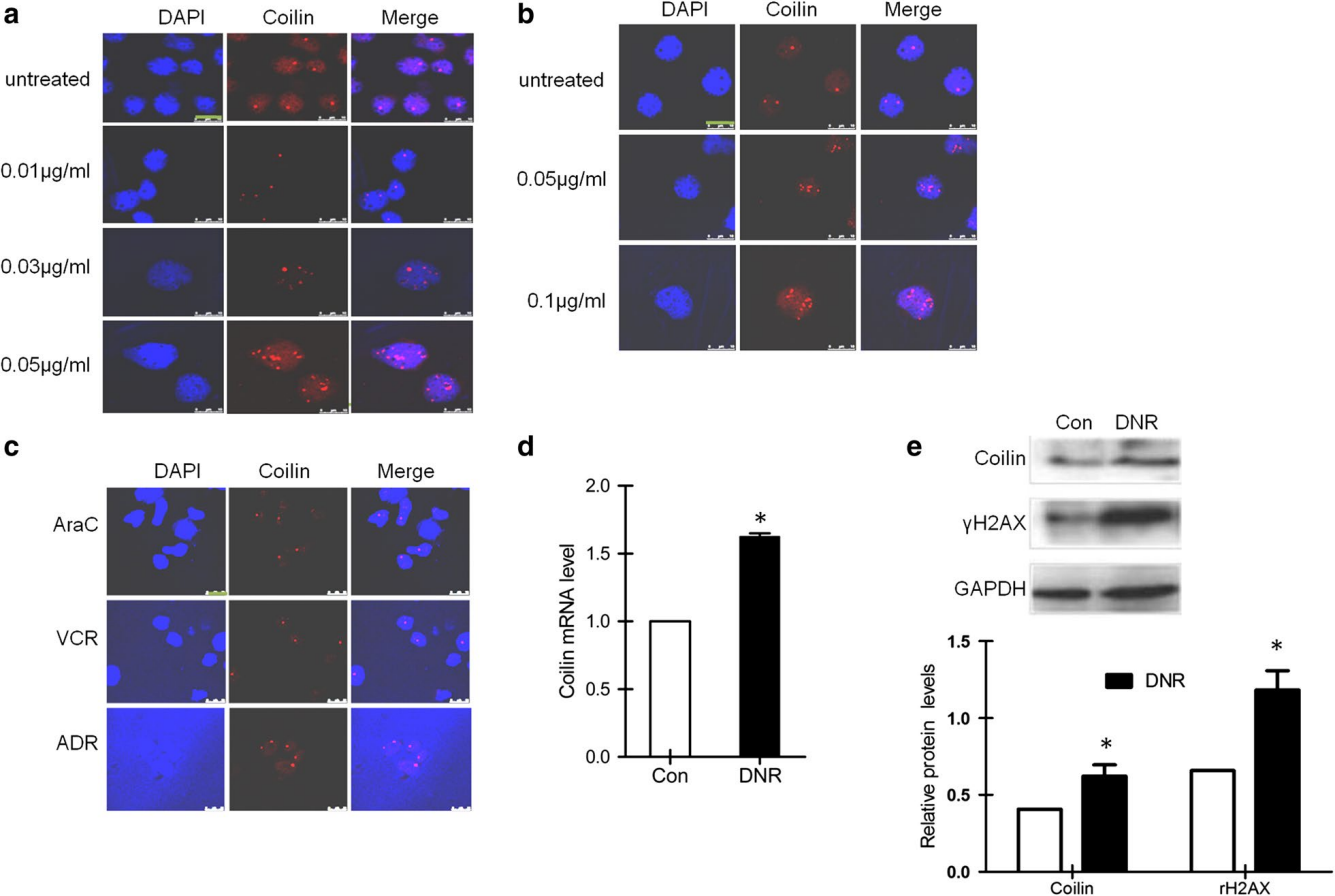
Subcellular localization and expression of coilin in leukemia cells following DNR treatments. Immunofluorescence of coilin in Reh (**a**) and RS4;11 (**b**) cells treated with different doses of DNR for 24 h. The nuclei were post-stained in blue with 4′,6-diamino-2-phenylindole (DAPI). **c** Immunofluorescence of coilin in Reh cells treated with arabinocytidine (AraC, 30 μg/ml), Vincristine (VCR, 1.5 μg/ml) or adriamycin (ADR, 2.0 μg/ml). **d** Coilin expression at the mRNA in Reh cells treated with DNR (0.03 μg/ml) for 24 h. Compared with the non-treated control, a significant increase (*p = 0.002, n = 3) was observed from the RT-qPCR measures. **e** Coilin expression at protein levels in Reh cells treated with DNR (0.03 μg/ml) for 24 h. Quantification from the Western blot of coilin (*p = 0.025) and γH2AX (*p = 0.003) also indicated increased levels normalized to GAPDH (n = 3)

Although the morphology coilin represented structures was known to respond to a variety of chemotherapy reagents, whether it can indicate the DNA damage responses in leukemia cells remained to be investigated. We first treated Reh cells with different doses of DNR and examined the localization of coilin by immunofluorescence assays (Fig. 1a). We found DNR treatments resulted in a drastic change in the localization of coilin, as well as its protein levels indicated by the intensity of staining. The coilin distribution appeared to be more scattered and partly concentrated toward the nucleolus. To exclude the possibility that the changes in coilin staining is cell-specific phenomenon in Reh, we performed similar experiments using another ALL cells, RS4;11 cells (Fig. 1b). We found a similar dislocation of coilin which was dose-dependent to DNR concentrations. To verify whether the changes in coilin staining following DNR treatments was indeed DNA damage associated, we compared the response to DNR with other types of chemicals of adriamycin (ADR), vincristine (VCR) and arabinocytidine (AraC) in Reh cells. We found that the redistribution of coilin could be observed in ADR treated cells, but not in cells exposed to VCR and AraC (Fig. 1c). Since ADR is the only agent known to cause massive DNA damages to a comparable extent in the experiment, the result indicated that the morphological changes of coilin was induced and correlated to DNA damages. Besides the morphology, we also performed RT-PCR and Western blotting for accurate measures of the relative expression levels of coilin following DNR treatments (Fig. 1d, e). Increased coilin expression at both mRNA and protein levels were found, consistent to the observation from IF staining. This prompted us to start exploring on whether DNR induced coilin expression could be observed or of clinical values from patient samples.

### Coilin expression exerted clinical values for correlating the outcome of pediatric BCP-ALL patients

**Fig. 2:**
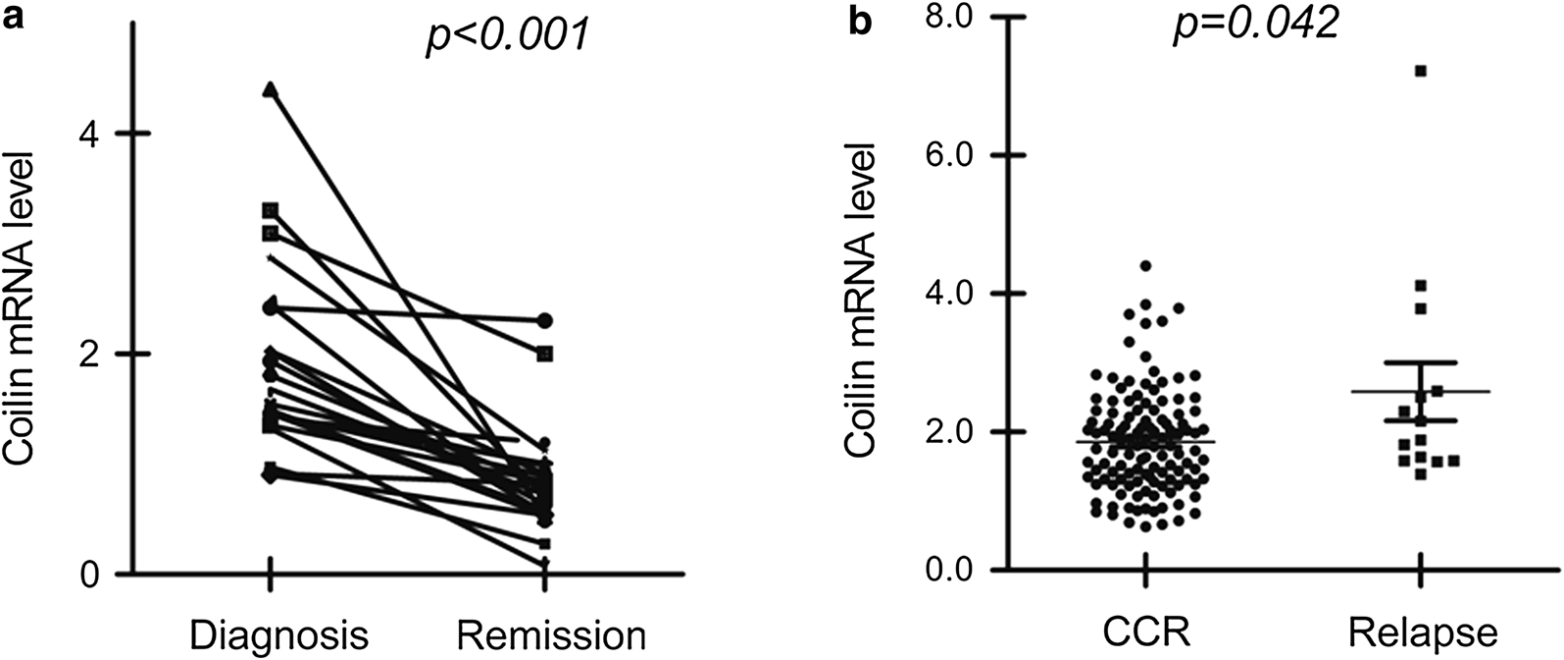
The coilin mRNA levels from the bone marrow samples in different clinical subgroups of pediatric ALL. **a** Relative coilin mRNA levels at diagnosis (day 0) or complete remission (day 78) (n = 22). **b** Coilin expression in patients of continuous complete remission (CCR) (n = 120) or disease relapse (n = 13)

A total of 144 patients with a first diagnosis of BCP-ALL were included in this study. The ratio of female to male was 1:1.48, with a median age of 4.1 ranging from 0.5 to 14.9 years. The 4-year event-free survival (EFS) was determined as 83.1 ± 3.1% after a median follow-up of 45 months (1.0–53.0). Of the 144 subjects, disease relapse occurred in 13 patients, including 12 cases of bone marrow (BM) relapse and 1 case of central nervous system (CNS) relapse. Eleven patients died from sepsis or multiple organ failure. The remaining 120 patients were in continuous complete remission (CCR), as shown in Table 1. The relative expression levels of the coilin mRNA were determined as between 0.63 and 7.2, with a median of 1.88. The expression of coilin with the value over 1.0 were observed in most of the samples as 88.2% (127/144 cases). With the exact paired 22 samples collected at day 0, the time of diagnosis, and day 78, in states of complete remission (CR), the result of coilin levels was summarized in Fig. 2a. Reduced coilin expression was found with a significance of p < 0.001. From the comparison between CCR and relapse patient groups (Fig. 2b), significant (p = 0.042) high expression of coilin was found in relapse patients (median 2.15, range 1.38–7.2; n = 13) over the CCR group (median 1.79, range 0.62–4.4; n = 120).

### Knockdown of coilin increased the DNR drug sensitivity in leukemia cells with the inhibition of p27 and the increase of apoptosis

**Fig. 3:**
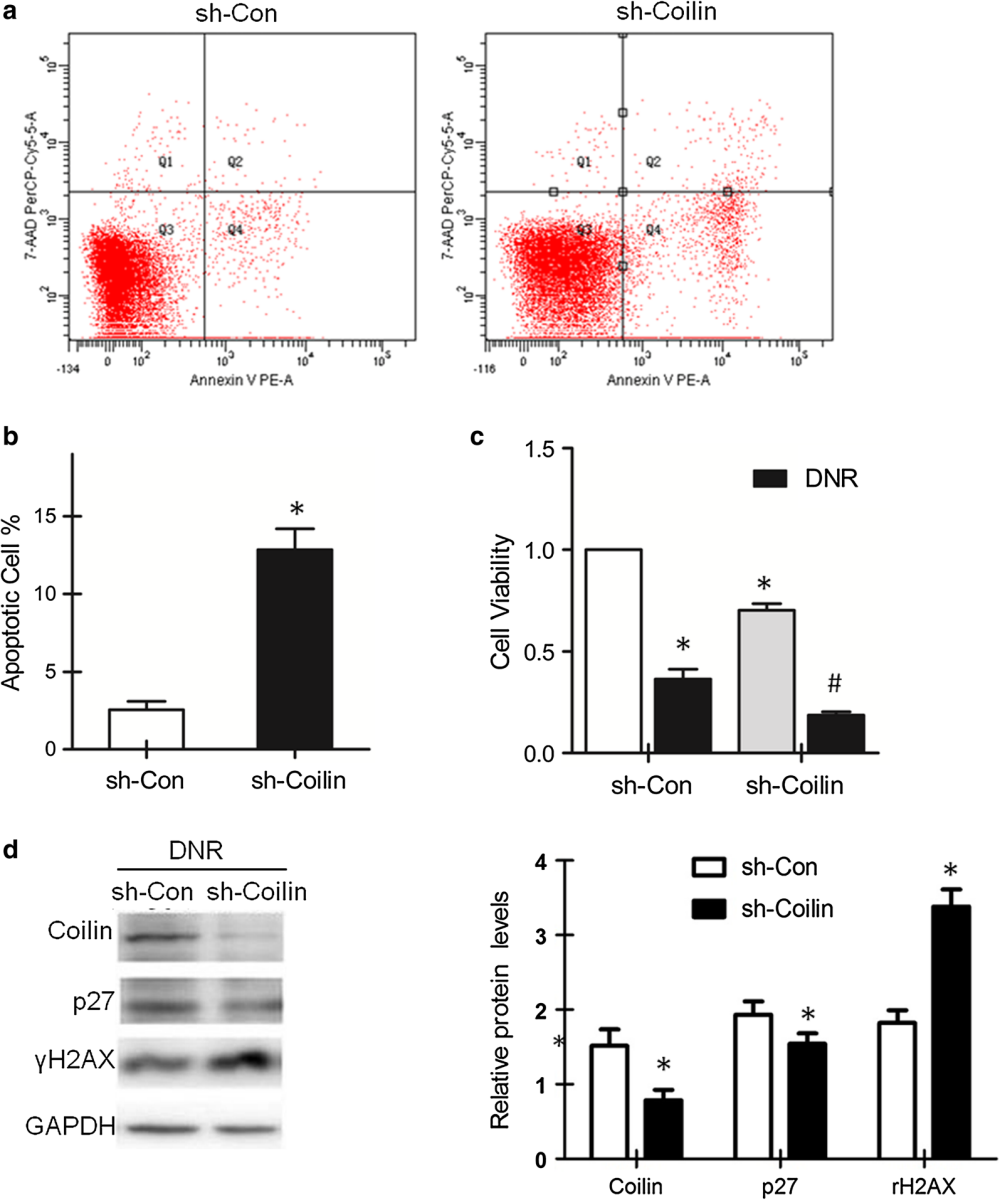
Effects of coilin knockdown in leukemia cells following DNR (0.03 μg/ml) treatments. **a** Flow cytometry analyses for apoptosis in Reh cells infected with a lenti-virus expressing a scrambled control or a shRNA targeting coilin. **b** Quantification on apoptosis (*p = 0.019, n = 3) from cells subjected to the same treatments as in **a**. **c** The cell survival measures in sh-Coilin infected Reh cells without or with DNR treatments (*p = 0.002, ^#^p = 0.014, n = 3). **d** Western blot of p27 and γH2AX from leukemia cells treated with DNR (0.03 μg/ml). Quantification (n = 3) of coilin, p27 and γH2AX proteins relative to GAPDH levels. The expression of coilin and p27 were decreased (*p = 0.009, p = 0.044); whereas γH2AX was increased (*p = 0.001)

From previous results, higher expression of coilin was found in ALL patients with poor prognosis. We wondered whether the expressed coilin proteins functionally involved in the attenuation of disease progression. We prepared a pair of lentivirus vector carrying either a control or a coilin shRNA expressing cassette to evaluate the knockdown effect of coilin in leukemia cells. Flow cytometry analysis was conducted with Reh cells infected with the lentiviruses. The results showed that significantly increased cell apoptosis was found in coilin suppressed cells as compared to the controls (Fig. 3a, b). We further examined whether coilin levels could affect sensitivity to DNR. When infected Reh cells were subjected to DNR for 24 h, MTS assays were performed to detect the survival rate of treated cells. As shown in Fig. 3c, sh-coilin infection reduced the live cell percentage to approximately 18% following DNR treatments, nearly as only a half of the values in the controls.

Besides apoptosis, cell cycle arrest could an alternative mechanism to cause significant reduction of survival cell numbers. As the result from apoptosis measures did not fully explain the survival cell data in percentage values, we began to exploit on regulatory molecules for cell cycle controls. One of the prominent candidate is the p27 protein. P27 is a potent inhibitor of various cyclin-CDK complexes that plays a critical roles in the cell correspond to DNA damages. Suppression of coilin induced elevated γH2AX levels following DNR treatment, as shown in the western blot (Fig. 3d). Infection of the lentivirus expressing sh-coilin, the reduction of p27 was observed in Reh cells, especially under conditions of DNR treatments. These results indicated that the expression coilin and p27 were coordinated for the determination of cell survival under DNR treatments.

### The prognostic value of coilin expression levels for treatment outcome and disease relapse in association with p27 in pediatric BCP-ALL patients

**Fig. 4:**
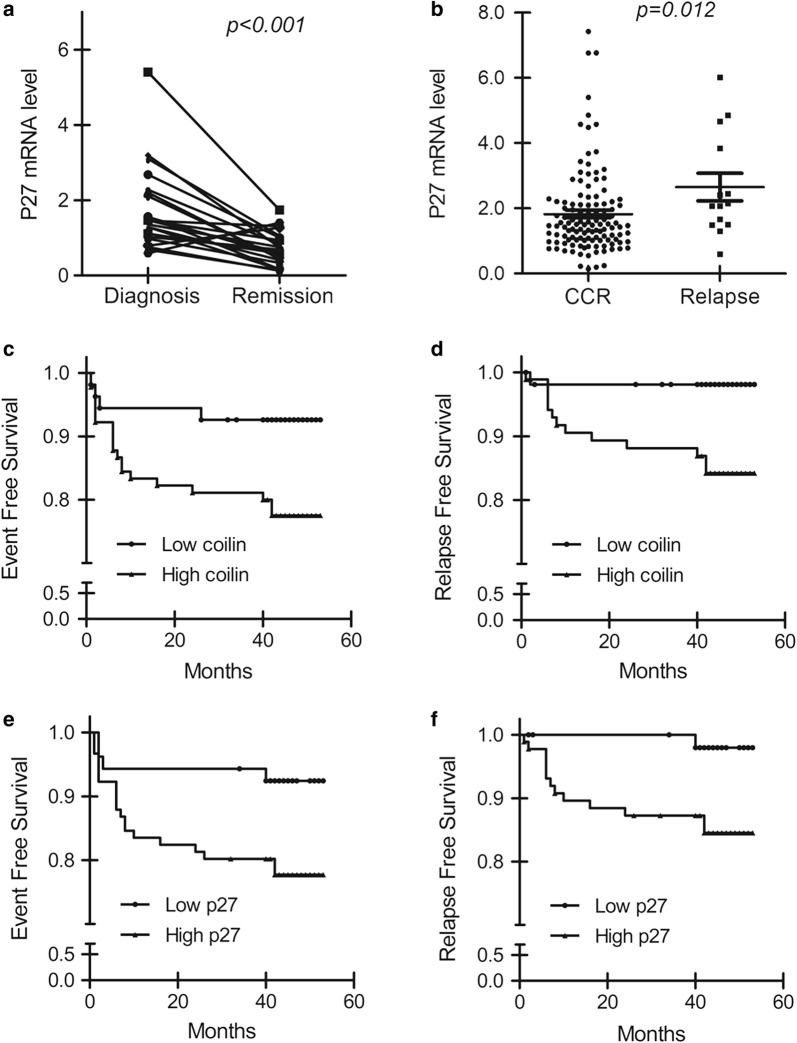
Expression of p27 in different subgroups conjoining coilin levels for the prognosis of pediatric BCP-ALL. **a** Relative p27 expression at diagnosis (day 0) or complete remission (day 78) (n = 22). **b** Levels of p27 expression in patients of continuous complete remission (CCR) (n = 120) or relapse (n = 13). Analyses of **c** event free survival (EFS) and **d** relapse free survival (RFS) from the whole cohort of 144 BCP-ALL patients with high or low coilin expression. Analyses of **e** event free survival (EFS) and **f** relapse free survival (RFS) in patients with high or low p27 expression

As we learned that the knockdown of coilin expression was able to clearly decreased the level of p27 significantly in cell cultures, especially under DNR treatment, we then started to examine the expression of p27 by RT-PCR in the 144 pediatric BCP-ALL patients. The relative expression levels of the p27 gene were between 0.14 and 7.42, with a median of 1.49. The levels of p27 mRNA with the value over 1.0 were found in 79.9% (115/144) cases. Similar to coilin, the mRNA levels of p27 at CR were reduced significantly in 20 cases among the 22 paired samples compared with that at diagnosis (p < 0.001, Fig. 4a). What’s more, the levels of p27 were higher in those patients who suffered relapse (n = 13; median 2.15, range 0.6–6.02) than patients in CCR (n = 120; median 1.46, range 0.14–7.42; p = 0.012; Fig. 4b). Notably, the expression of coilin and p27 appeared to be correlated with Spearman correlation coefficient of 0.421 (p < 0.001).

As the expression of p27 has been reported to regulate apoptosis and cell survival and was suggested for the prognosis in AML and CLL. We analyzed the survivals of the BCP-ALL patients in correlation with the levels of p27. The threshold of p27 measures was determined by ROC curve analysis, and the optimal cut-off level of p27 was 1.30. The whole cohort was separated into high- and low-p27 expression groups of 91 and 53 cases respectively by the cut-off value. The 4-year EFS and RFS were poorer in high-p27 group as determined to be 77.7 ± 4.4 and 85.5 ± 3.9% than those patients in low-p27 group (92.4 ± 3.6, 98.0 ± 2.0%) with statistical significance of p = 0.028 and p = 0.02 (Fig. 4e, f). Similarly, the threshold of coilin expression of a cut-off level as 1.57 was used to define high- and low-coilin expression groups of 90 and 54 cases. As shown in Fig. 4c, d, low coilin group displayed a significant favorable result on EFS and RFS measures (92.6 ± 3.6, 98.1 ± 1.9%). These results suggested that high expression of the coilin and p27 gene was not only potentially indicated non-optimistic drug responses in leukemia therapies, but also could be used for predicting poor treatment outcome.

## Discussion

Earlier studies seemed to suggest that the morphology of Cajal bodies appeared to be more dynamic organelle in higher metabolic cells, such as neurons or cancer cells. The number and sizes of CBs were affected with changes in cell cycle, development, transformation, temperature, DNA damage, and coilin mutations [23]. The phosphorylation of coilin at different amino acid residues triggered the formation or destabilization of CBs. Several latest studies have demonstrated the redistribution of coilin responded to DNA damages in solid tumor cells [24]. It will be interesting to learn the morphology and changes in leukemia cells, where a variety of DNA damage agents were used for the clinical treatments for the disease. However, very few reports were found to this aspect. In this study, we treated Reh and RS4;11 cells with DNR of various concentrations and discovered dramatical changes in CB morphology, particularly the relocalization of coilin into the nucleolus. Similar phenomenon was seen during which we treated the cells with ADR, an analog of DNR as another DNA damaging agent, a dose-dependent change in CB morphology was observed similar to the cells following DNR treatments. To make sure that the changes in CB and coilin were indeed the consequence of DNA damages, we also treated the cells with other agents used in leukemia therapies of AraC or VCR. AraC belongs to the pyrimidine class of anti-metabolic drugs; whereas VCR is known to bind tubulin and cause mitosis arrest in the meta-phase. As shown in Fig. 1c, the results were clearly different in the microscopic pictures.

The drug-responsive morphological changes of CBs was also demonstrated to relate to DNA repair processes. It was reported that Herpes virus infection redistributed coilin to the damaged centromeres by activating the interphase centromere damage response (iCDR) [25]. In addition, UV-C exposure disrupted CB requires the PA28 interactions with the nucleoplasmic pool of coilin [26]. DNR and ADR induced DNA damage triggered the nucleolar accumulation of coilin, as observed in this study, also suggested the DNA repair machinery was activated. Although the exact cause and the molecular interactions for inducing the changes in CB formation were not clear, it was generally accepted that coilin might directly involved in the cellular stress response pathways, especially certain nuclear events under conditions of drug treatment. In fact, coilin knockdown promoted apoptosis and increased chemo-sensitivity to DNR as demonstrated from our experiments, provided supportive evidence of this notion.

Interestingly, suppressing of coilin expression sufficiently caused a significant reduction of p27 levels under DNR treatment. This seemed to be consistent with the finding that the expression of coilin and p27 were notably correlated at the mRNA levels in BCP-ALL patients. The principal function of p27 is to regulate cell cycle progression, the effects on cell survival and apoptosis appeared to be very much context dependent under drug treatments. Some studies have reported that p27 might negatively regulate apoptosis, more researches showed p27 induced apoptosis [27]. The outcome for p27 overexpression on cell survival was also appeared to be cell-type specific [28]. The subcellular localization of p27 was suggested as an important factor for consideration in solid tumor cells, it is less relevant in our study when leukemia cells were being used. Although from this study, the effect of coilin knockdown for inducing apoptosis and increasing the daunorubicin sensitivity was demonstrated, as well as the involvement of p27 was suggested, further investigation to reveal the underlining mechanisms was in need and could be important for clinical practice.

The advance for ALL treatment has significantly improved the patient outcome. As in pediatric leukemia where higher doses of chemotherapy drugs were often applied, better remission in CCR rate can be achieved. However, there are currently no satisfactory makers for the prognosis of disease relapse, even at the time of recurrence [29]. From the investigation using BCP-ALL patient BM samples, we found that most of the remission patients following treatments exerted reduced levels of coilin and p27 expression comparing to the time at initial diagnosis. Meanwhile, the coilin and p27 expressions in the relapsed patients were significantly higher compared with the continuous CR patients. The significant p value from the statistics indicated that high expression of coilin and p27 could be a marker for poor drug response and an indicator high incidence of relapse. High levels of snRNP assembly and pre-mRNA splicing was frequently observed in tumor cells and contributed in the increase of cell viability [30, 31]. As coilin is a major constituent protein of CB where snRNP species are being processed, it is reasonable to expect reduced coilin levels favored a better prognosis. With the additional discovery that coilin was able to modulate the expression of p27, it explained, at least partially the suppressive effect of coilin knockdown on cell survival amplified under DNR treatment.

Although p27 negatively controls cell-cycle progression and has been classically considered as a tumor suppressor, many evidences indicated that it may have oncogenic property as well. The cytoplasmic localized p27 was observed to associate with poor prognosis in AML and CML [27, 32]. Interestingly, it was contradictory about the effects of p27 expression level on prognosis in leukemia. Radosevic reported that high p27 protein expression was found in the newly diagnosed AML and patients who have higher p27 protein expression were more sensitive to chemotherapy [33]. However Haferlach reported that the low level of p27 mRNA was a good indicator of prognosis in AML [22]. This may be due to different subtypes and treatment protocols of patients. In this study, we performed a conjoint analysis on the prognostic significance of coilin and p27. Similar to Haferlach et al.’s findings, our results indicated that low expression of coilin and p27 indicated low recurrence. Survival analysis also confirmed that the estimated 4-year EFS and RFS in low-p27 were much higher than those in high-p27 group.

## Conclusion

In summary, we observed that daunorubicin, one of the most commonly used chemotherapeutic drugs in ALL, induced coilin relocated to the nucleolus. Reducing of the expression of coilin induced apoptosis, increased the sensitivity to daunorubicin and repressed the expression of p27 in Reh cells. In pediatric BCP-ALL patients, low expression of coilin and p27 mRNA were found in complete remission ALL patients compared with the control group. Therefore, our observations provided a new strategy that combined detection of the mRNA of coilin and p27 could to help predict the recurrence and prognosis of pediatric ALL.

The morphology of Cajal bodies as indicated by coilin can be easily detected by immunofluorescence analyse as a routine examination. With the rapid progress in the development and application of high-content clinical microscopic equipment, the examination of coilin or perhaps other marker proteins of CBs could be used to form a big data digital source to allow cumulative research for leukemia treatments. With the facilitation of advanced imaging and analysis software, the process can be highly automatic, and potentially also give the readout for semi-quantitative measures of gene expression levels, for examples, from the staining intensity of coilin. In clinics, the patient peripheral blood smear can be conveniently obtained and archived for storage, this will make retrospective research to search and establish indexes of diagnosis or prognosis for the evaluation of disease progression and recurrence. In ALL leukemia, the less irregular shapes of malignant cells in nature as compared to other solid tumor types will be a giant advantage to permit the very first attempt of these novel strategies.

## Abbreviations

ALL: acute lymphoblastic leukemia
CB: cajal body
p27: cyclin-dependent kinase inhibitor 1B
CR: complete remission
DNR: daunorubicin
FISH: fluorescence in situ hybridization
EFS: event-free survival
OS: overall survival

## References

[1] Gao C, Zhang RD, Liu SG, Zhao XX, Cui L, Yue ZX, Li WJ, Chen ZP, Li ZG, Rao Q, Wang M, Zheng HY, Wang JX (2017). Low CREBBP expression is associated with adverse long-term outcomes in paediatric acute lymphoblastic leukaemia. Eur J Haematol.

[2] Antillon FG, Blanco JG, Valverde PD, Castellanos M, Garrido CP, Giron V, Letona TR, Osorio EJ, Borrayo DA, Mack RA, Melgar MA, Lorenzana R, Ribeiro RC, Metzger M, Conter V, Rossi E, Valsecchi MG (2016). The treatment of childhood acute lymphoblastic leukemia in Guatemala: biologic features, treatment hurdles, and results. Cancer.

[3] Cui L, Gao C, Zhang RD, Jiao Y, Li WJ, Zhao XX, Liu SG, Yue ZX, Zheng HY, Deng GR, Wu MY, Li ZG, Jia HT (2015). Low expressions of ARS2 and CASP8AP2 predict relapse and poor prognosis in pediatric acute lymphoblastic leukemia patients treated on China CCLG-ALL 2008 protocol. Leuk Res.

[4] Zhang H, Cheng H, Wang Q, Zeng X, Chen Y, Yan J, Sun Y, Zhao X, Li W, Gao C, Gong W, Li B, Zhang R, Nan L, Wu Y, Bao S, Dong J, Han J, Zheng H (2015). An advanced fragment analysis-based individualized subtype classification of pediatric acute lymphoblastic leukemia. Sci Rep..

[5] Moorman AV, Enshaei A, Schwab C, Wade R, Chilton L, Elliott A, Richardson S, Hancock J, Kinsey SE, Mitchell CD, Goulden N, Vora A (2014). Harrison CJ.A novel integrated cytogenetic and genomic classification refines risk stratification in pediatric acute lymphoblastic leukemia. Blood.

[6] Cui L, Li Z, Wu M, Li W, Gao C, Deng G (2010). Combined analysis of minimal residual disease at two time points and its value for risk stratification in childhood B-lineage acute lymphoblastic leukemia. Leuk Res.

[7] Cowell IG, Austin CA (2012). Do transcription factories and TOP2B provide a recipe for chromosome translocations in therapy-related leukemia?. Cell Cycle..

[8] Pogorzala M, Kubicka M, Rafinska B, Wysocki M, Styczynski J (2015). Drug-resistance profile in multiple-relapsed childhood acute lymphoblastic leukemia. Anticancer Res.

[9] Housman G, Byler S, Heerboth S, Lapinska K, Longacre M, Snyder N, Sarkar S (2014). Drug resistance in cancer: an overview. Cancers Basel..

[10] Delgado JL, Hsieh CM, Chan NL, Hiasa H (2018). Topoisomerases as anticancer targets. Biochem J..

[11] Lafarga M, Tapia O, Romero AM, Berciano MT (2016). Cajal bodies in neurons. RNA Biol.

[12] Cioce M, Lamond AI (2005). Cajal bodies: a long history of discovery. Annu Rev Cell Dev Biol..

[13] Machyna M, Neugebauer KM, Stanek D (2015). Coilin: the first 25 years. RNA Biol..

[14] Bartova E, Foltankova V, Legartova S, Sehnalova P, Sorokin DV, Suchankova J, Kozubek S (2014). Coilin is rapidly recruited to UVA-induced DNA lesions and gamma-radiation affects localized movement of Cajal bodies. Nucleus..

[15] Velma V, Carrero ZI, Cosman AM, Hebert MD (2010). Coilin interacts with Ku proteins and inhibits in vitro non-homologous DNA end joining. FEBS Lett.

[16] Vogan JM, Zhang X, Youmans DT, Regalado SG, Johnson JZ, Hockemeyer D, Collins K (2016). Minimized human telomerase maintains telomeres and resolves endogenous roles of H/ACA proteins, TCAB1, and Cajal bodies. Elife..

[17] Xu H, Pillai RS, Azzouz TN, Shpargel KB, Kambach C, Hebert MD, Schumperli D, Matera AG (2005). The C-terminal domain of coilin interacts with Sm proteins and U snRNPs. Chromosoma.

[18] Machyna M, Kehr S, Straube K, Kappei D, Buchholz F, Butter F, Ule J, Hertel J, Stadler PF, Neugebauer KM (2014). The coilin interactome identifies hundreds of small noncoding RNAs that traffic through Cajal bodies. Mol Cell.

[19] Velma V, Carrero ZI, Allen CB, Hebert MD (2012). Coilin levels modulate cell cycle progression and gammaH2AX levels in etoposide treated U2OS cells. FEBS Lett.

[20] Roy A, Banerjee S (2015). p27 and leukemia: cell cycle and beyond. J Cell Physiol..

[21] Caraballo JM, Acosta JC, Cortes MA, Albajar M, Gomez-Casares MT, Batlle-Lopez A, Cuadrado MA, Onaindia A, Bretones G, Llorca J, Piris MA, Colomer D, Leon J (2014). High p27 protein levels in chronic lymphocytic leukemia are associated to low Myc and Skp2 expression, confer resistance to apoptosis and antagonize Myc effects on cell cycle. Oncotarget..

[22] Haferlach C, Bacher U, Kohlmann A, Schindela S, Alpermann T, Kern W, Schnittger S, Haferlach T (2011). CDKN1B, encoding the cyclin-dependent kinase inhibitor 1B (p27), is located in the minimally deleted region of 12p abnormalities in myeloid malignancies and its low expression is a favorable prognostic marker in acute myeloid leukemia. Haematologica.

[23] Trinkle-Mulcahy L, Sleeman JE (2016). The cajal body and the nucleolus: “In a Relationship” or “It’s Complicated”?. RNA Biol..

[24] Gilder AS, Do PM, Carrero ZI, Cosman AM, Broome HJ, Velma V, Martinez LA, Hebert MD (2011). Coilin participates in the suppression of RNA polymerase I in response to cisplatin-induced DNA damage. Mol Biol Cell.

[25] Sabra M, Texier P, El Maalouf J, Lomonte P (2013). The Tudor protein survival motor neuron (SMN) is a chromatin-binding protein that interacts with methylated lysine 79 of histone H3. J Cell Sci.

[26] Tapia O, Bengoechea R, Palanca A, Arteaga R, Val-Bernal JF, Tizzano EF, Berciano MT, Lafarga M (2012). Reorganization of Cajal bodies and nucleolar targeting of coilin in motor neurons of type I spinal muscular atrophy. Histochem Cell Biol.

[27] Agarwal A, Mackenzie RJ, Besson A, Jeng S, Carey A, LaTocha DH, Fleischman AG, Duquesnes N, Eide CA, Vasudevan KB, Loriaux MM, Firpo E, Cortes JE, McWeeney S, O’Hare T, Roberts JM, Druker BJ, Deininger MW (2014). BCR-ABL1 promotes leukemia by converting p27 into a cytoplasmic oncoprotein. Blood.

[28] Bencivenga D, Caldarelli I, Stampone E, Mancini FP, Balestrieri ML, Della Ragione F, Borriello A (2017). p27(Kip1) and human cancers: a reappraisal of a still enigmatic protein. Cancer Lett.

[29] Wang KL, Mei YY, Cui L, Zhao XX, Li WJ, Gao C, Liu SG, Jiao Y, Liu FF, Wu MY, Ding W, Li ZG (2014). E2F3a gene expression has prognostic significance in childhood acute lymphoblastic leukemia. Eur J Haematol.

[30] Enwerem II, Velma V, Broome HJ, Kuna M, Begum RA, Hebert MD (2014). Coilin association with Box C/D scaRNA suggests a direct role for the Cajal body marker protein in scaRNP biogenesis. Biol Open..

[31] Song Y, Niu J, Yue Z, Gao R, Zhang C, Ding W (2017). Increased chemo-sensitivity by knockdown coilin expression involved acceleration of premature cellular senescence in HeLa cells. Biochem Biophys Res Commun.

[32] Roy A, Lahiry L, Banerjee D, Ghosh M, Banerjee S (2013). Increased cytoplasmic localization of p27(kip1) and its modulation of RhoA activity during progression of chronic myeloid leukemia. PLoS ONE.

[33] Radosevic N, Delmer A, Tang R, Marie JP, Ajchenbaum-Cymbalista F (2001). Cell cycle regulatory protein expression in fresh acute myeloid leukemia cells and after drug exposure. Leukemia.

